# Mushroom Poisoning

**Published:** 1913-05-15

**Authors:** 


### MUSHROOM POISONING.

Poisoning by this fungus is not at all infrequent, and consequently the treatment of this condition is worthy of careful consideration. In this country edible mushrooms found growing wild are gathered much more frequently than they used to be, both because of the example set by our foreign-born residents, and because of the greater demand for them as a delicacy in hotels and restaurants. Mushrooms are also cultivated, and a certain variety brings a good price. It is perhaps rare for the poisonous varieties of mushroom to be dispensed by a hotel or restaurant, the greatest care being exercised in deciding that the product offered is edible, that is, non-poisonous; but children sometimes obtain and eat the poisonous mushroom, and private families occasionally cook and serve it.

The mushroom is rich in nitrogen, but it is uncertain how much of this nitrogen can be utilized as a food; it is said to be absorbed only in small part, and is likely, during its digestion, to cause some gastro-intestinal irritation in many persons. As a nutrient the mushroom is of little value, since a large proportion of it is water.

Over two thousand varieties of mushrooms have been described, some of which are harmless, and others exceedingly poisonous. Gibson[1] considers that the usual methods of distinguishing between edible and poisonous varieties are unreliable, and suggests the following considerations in the determination as to their value: "1. Avoid every mushroom having a cup, or suggestion of such, at its base; the distinctly fatal poisons are thus excluded. 2. Exclude those having an unpleasant odor, a peppery, bitter or other unpalatable flavor, or tough consistency." The *Amanita muscaria* and the *Amanita phalloides* are regarded as among the most poisonous, and contain the active principle muscarin or amanitin. The *Amanita muscaria* is "distinguished by its bright-orange or red pileus (cap) covered with soft, fugacious, whitish warts, its white, rarely yellowish lamellae (gills), and its white floccose stipes (stem), bulbous at the base and bearing, at its upper attenuated extremity, a white ring."[2] The common edible mushroom is the *Agaricus campestris*, belonging to the subvariety of *Psalliota*, "distinguished by the silky floccose or finely scaly pileus, the somewhat reddish tint of the flesh, and the pinkish lamellae changing to a dark brown. There are many varieties, some of which are cultivated on a large scale, especially in France."[2]

All fungi after being picked are prone to rapid decomposition. Some of the poisonous constituents in edible mushrooms due to decomposition, and some of the poisonous constituents of non-edible fungi may be destroyed or dispersed by heat, especially if thoroughly cooked. Many of the products of decomposition, however, and the poisons in poisonous mushrooms are irritant to the gastro-intestinal tract and poisonous to the nervous system. Some persons are more or less seriously poisoned even by edible mushrooms that are not fresh, and there are persons who have an idiosyncrasy for all mushrooms or for certain varieties.

---

1. Gibson, W. Hamilton: Our Edible Toadstools and Mushrooms. Harper & Brothers, 1903, p. 33.

2. Foster, Frank P.: Medical Dictionary, under Agaricus, i, 108.

The alkaloidal poison of the *Amanita muscaria*, termed muscarin, closely simulates in its molecular constitution cholin, which is a constituent of some animal tissues, and under certain conditions is set loose in the intestine and acts as a nervous irritant. This alkaloid muscarin, which is the constituent of non-edible mushrooms that causes poisoning, resembles in its physiologic action pilocarpin, although it is very much more active poison.

After one or more poisonous mushrooms have been eaten, rarely will there be nausea and vomiting within a short time. Generally the absorption of the toxin does not take place until after the mushrooms have entered the intestine. Symptoms of poisoning do not occur for several hours after the mushrooms have been eaten; then pain, cramps, reflex vomiting and watery purging are likely to occur. If much of the poisoning has been absorbed before this local irritation takes place, symptoms of central poisoning occur, as noted by quickened pulse, rapid respiration, dizziness, muscular tremblings, and perhaps signs of intoxication bordering on confusion and delirium. The pupils are contracted. If the poisoning is more serious, the respiration and heart fail and the patient may die in collapse, consciousness generally not being lost. As in pilocarpin poisoning, there is a more or less profuse perspiration, and a free flow of saliva and bronchial mucus. This profuse perspiration and the watery stools cause great thirst and prostration.

If for any reason an insufficient amount of the poisonous mushroom has been ingested to cause gastro-intestinal irritation, before purging begins a sufficient amount of the toxin may have been absorbed to cause the nervous symptoms to be the most important and prominent. This kind of poisoning does not occur until eight or ten hours after the ingestion of the mushrooms. In this form of poisoning the most troublesome symptoms are vertigo, disturbed vision, irregular and weak pulse, and muscle twitchings or actual rigidity. The extremities become cold and the face becomes pale or bluish; there may be collapse, and later semiparalysis. In these instances of late poisoning there may be drowsiness and stupor as well as intense dyspnea. A greatly impaired circulation may be the cause of this cerebral condition, as coma is probably not a part of true muscarin poisoning. When the symptoms of poisoning appear late, as just described, showing that a large amount of the toxin has been absorbed before vomiting or purging has protected the system, the prognosis is much more serious.

The treatment of mushroom poisoning depends somewhat on the time when the symptoms occur. If they occur soon after the ingestion of the mushroom, a brisk emetic is the first treatment; the choice of an emetic, whether zinc sulphate, ipecac or mustard water, is a matter of unimportance, provided that the stomach is emptied. As soon as the stomach is emptied by the emetic, or by vomiting without the aid of an emetic, large draughts of hot water or flaxseed tea, slippery elm tea or starch water should be given to wash off and sooth the mucous membrane. As soon at the stomach is quiet and the vomiting has ceased, a cathartic should be given; none is better than castor oil, as it is stated that an oil does not dissolve the toxic principle or promote its absorption. An ordinary dose of castor oil would be 30 c.c. (an ounce), given in the manner the least disagreeable to the patient. If the oil is not retained long enough to have passed into the intestine, a saline, as magnesium sulphate or sodium sulphate, may be given.

Alcohol in any form should probably not be given, as it may promote the absorption of the toxin. Strong coffee, however, is indicated. Hot, dry applications to the body, friction of the extremities and a hot-water bag at the feet tend to prevent shock and depression from this nerve poison.

The physiologic antidote to muscarin is atropin, which should be administered immediately, hypodermically; 1-100 grain should be given, perhaps combined with 1-30 grain of strychnin, both of which drugs act also as circulatory stimulants. If the collapse is prolonged, the strychnin may be repeated within three or four hours. The atropin may be repeated in five or six hours, and to offset a prolonged collapse, some form of digitalis may be given, either by the mouth or hypodermically. It should be remembered that digitalis acts slowly, and will be of little value for from six to ten hours. The value of camphor, given subcutaneously, as a cerebral and circulatory stimulant should be remembered. The contents of an ampule containing a saturated solution of camphor in sterile oil should be administered subcutaneously every hour for several doses, if needed. The coffee, or caffein, should also be repeated.

Death may occur after two or three days from mushroom poisoning; therefore, even with apparent improvement, the patient should be kept at rest and all care be taken that sudden collapse does not occur.

If the vomiting persists, or the diarrhea becomes too profuse and continuous, the sedative and soothing action of bismuth subcarbonate should be utilized, and soothing, mucilaginous warm drinks should be given. A bland, warm cereal should be the first food given. As previously stated, the patient is likely to be thirsty and crave cold liquids, but it is better that he receive warm liquids.

If the vomiting becomes serious, or the purging is not tractable, and especially if there is severe pain, a hypodermatic injection of a small dose of morphin should be given. The liquids taken, either on account of thirst or for sedative purposes or as foods, soon promote elimination by the kidneys, especially if the blood-pressure has been improved and collapse is not in evidence.

The prognosis is usually good in mushroom poisoning, though the patients are very ill for a few hours or days.—*Journal A. M. A.*

